# A Systematic Review and Meta-Analysis of Herbal Medicine on Chronic Obstructive Pulmonary Diseases

**DOI:** 10.1155/2014/925069

**Published:** 2014-03-26

**Authors:** Hai Yong Chen, Chun Ho Ma, Ke-Jian Cao, James Chung-Man Ho, Eric Ziea, Vivian Taam Wong, Zhang-Jin Zhang

**Affiliations:** ^1^School of Chinese Medicine, LKS Faculty of Medicine, The University of Hong Kong, 10 Sassoon Road, Pokfulam, Hong Kong; ^2^Department of Medicine, LKS Faculty of Medicine, The University of Hong Kong, Pokfulam, Hong Kong; ^3^Chinese Medicine Section, Hospital Authority, Kowloon, Hong Kong

## Abstract

Herbal medicine (HM) as an adjunct therapy has been shown to be promising for the chronic obstructive pulmonary disease (COPD). However, the role of herbs in COPD remains largely unexplored. In this present study, we conducted the systematic review to evaluate the efficacy of herbs in COPD. 176 clinical studies with reporting pulmonary function were retrieved from English and Chinese database. Commonly used herbs for acute exacerbations stage (AECOPD) and stable COPD stage (SCOPD) were identified. A meta-analysis conducted from 15 high quality studies (18 publications) showed that HM as an adjunct therapy had no significant improvement in pulmonary function (FEV_1_, FEV%, FVC, and FEV_1_/FVC) compared to conventional medicine. The efficacy of the adjunct HM on improving the arterial blood gas (PaCO_2_ and PaO_2_) for AECOPD and SCOPD remains inconclusive due to the heterogeneity among the studies. However, HM as an adjunct therapy improved clinical symptoms and quality of life (total score, activity score, and impact score of St. George's Respiratory Questionnaire). Studies with large-scale and double-blind randomized controlled trials are required to confirm the role of the adjunct HM in the management of COPD.

## 1. Introduction

Chronic obstructive pulmonary disease (COPD) is a progressive and chronic lung disease. It is the fourth leading cause of death as reported by the World Health Organization. COPD is characterized by chronic cough, excess sputum production, chest tightness, and shortness of breath during physical activity [1]. In the European Union, the total direct costs of respiratory diseases are estimated to be about 6% of the total health care budget, with COPD accounting for 56% of the cost under respiratory diseases [2]. Studies have predicted that the rank of disability adjusted life years (DALYs) in COPD will rise from the seventh to fifth by 2030 [3, 4]. Though stable COPD (SCOPD) and acute exacerbations (AECOPD) are treatable [5–9], there is no cure. Studies have shown the promising herbal medicine (HM) (including Chinese medicine and medicinal plant or its derivatives from other countries) in relieving COPD by attenuating disease symptoms, improving quality of life and pulmonary function [10–13], and reducing the frequency, duration, and severity of exacerbations [14]. Emerging studies also demonstrated the anti-inflammatory and antioxidative effect of HM in COPD disease [10, 15–17].

Recently, several systematic reviews have been conducted, indicating the potential effectiveness of Chinese herbal medicine in COPD [18–25]. However, the efficacy remains inconclusive due to low quality of studies. Pulmonary function has been considered the gold standard to the diagnosis of COPD. In the present study, we systematically reviewed the efficacy of HM on COPD using the pulmonary function as the primary outcome. In addition, the commonly used herbs and secondary outcome such as quality of life and clinical symptoms were analyzed.

## 2. Methods

### 2.1. Search Strategy

Search strategy used in this study was as follows: (Chinese medicine OR Traditional Chinese Medicine OR Chinese herbal medicine OR Chinese herbal drug OR traditional herbal medicine OR herbal medicine OR traditional Japanese medicine OR traditional medicine OR Ethnomedicine OR Folk Medicine OR Folk Remedies OR Home Remedies OR Indigenous Medicine OR Primitive Medicine OR Materia Medica OR Homeopathic Remedies OR Nosodes OR traditional East Asian medicine OR Traditional Far Eastern Medicine OR Far East Medicine OR Oriental medicine OR Korean medicine OR Tibetan medicine OR herb OR herbaceous agent OR medicinal plant OR medicinal herbs OR medicinal plant product OR plant extract OR plant preparation OR Herbal Preparation OR botanic OR botany OR Kampo OR Kampo medicine OR Kanpo OR Traditional Mongolian Medicine OR Mongolian Folk Medicine OR Mongolian Medicine OR phytotherapy OR Herb Therapy OR Herbal Therapy OR Ethnopharmacology OR Ethnobotany OR phytogenics OR alternative medicine OR Alternative Therapy OR complementary therapy OR Complementary Medicine OR TCM OR CHM OR Zhong Yi Xue) AND (Chronic obstructive pulmonary disease OR COPD OR chronic obstructive lung disease OR chronic obstructive airway disease OR chronic airflow limitation OR chronic obstructive respiratory disease OR chronic bronchitis OR chronic emphysema OR shortness of breath OR SOB OR dyspnea). The keywords search was conducted in March 2012 and updated in November 2013.

### 2.2. Database

Search strategy followed the Cochrane Collaboration without restriction of the language for publications. The following databases were searched: (1) PubMed; (2) MEDLINE; (3) EMBASE; (4) Cochrane Central Register of Controlled Trials (CENTRAL); (5) Cochrane Database of Systematic Reviews; (6) AMED; (7) China Academic Journal Full Text Database; and (8) China Master and Doctor Theses Full-Text Database.

### 2.3. Inclusion Criteria

Inclusion criteria included all published studies reporting quasi or randomized controlled trials (RCTs) and comparing HM or its variants as monotherapy or adjuvant therapy, with at least one control group that used the conventional western practice or placebo. Participants were required to have had a diagnosis of COPD according to the investigators of the trials. There would be no restriction on the ethnicity, gender, age, or disease duration of the participants in the trials. The HM interventions could have been either (1) extract(s) from a single herb; (2) preparation(s) containing multiple herbs; or (3) proprietary herbal product(s). They should be used alone or coadministered with western medications. The control intervention has to be either a placebo or a western medication (WM). Trials of all durations are welcomed. Both parallel and crossover designs would be accepted. Clinical studies had to report the pulmonary function in the research.

### 2.4. Exclusion Criteria

Studies without baseline and endpoint of pulmonary function tests during clinical trials were excluded.

### 2.5. Data Synthesis

All analyses were performed with RevMan version 5.1 (The Nordic Cochrane Centre, The Cochrane Collaboration) to quantify and compare the efficacy of outcomes of the treatment versus the control groups. Standard mean difference was given for continuous outcome variable with 95% confidence interval (CI), while odds ratio (OR) was given for dichotomous outcome variable with 95% CI. The random-effect model was employed when the study of heterogeneity (*I* ^2^) was larger than 50%; otherwise a fix-effect model was used when the* I* ^2^ was less than 50%. To test the heterogeneity, subgroup analysis was performed according to the AECOPD and SCOPD. The *Z* test was used to compare the overall effects of the treatment groups and the control groups; differences were considered to be statistically significant when *P* < 0.05.

## 3. Results

### 3.1. Characterization of Studies Included

**Figure 1:**
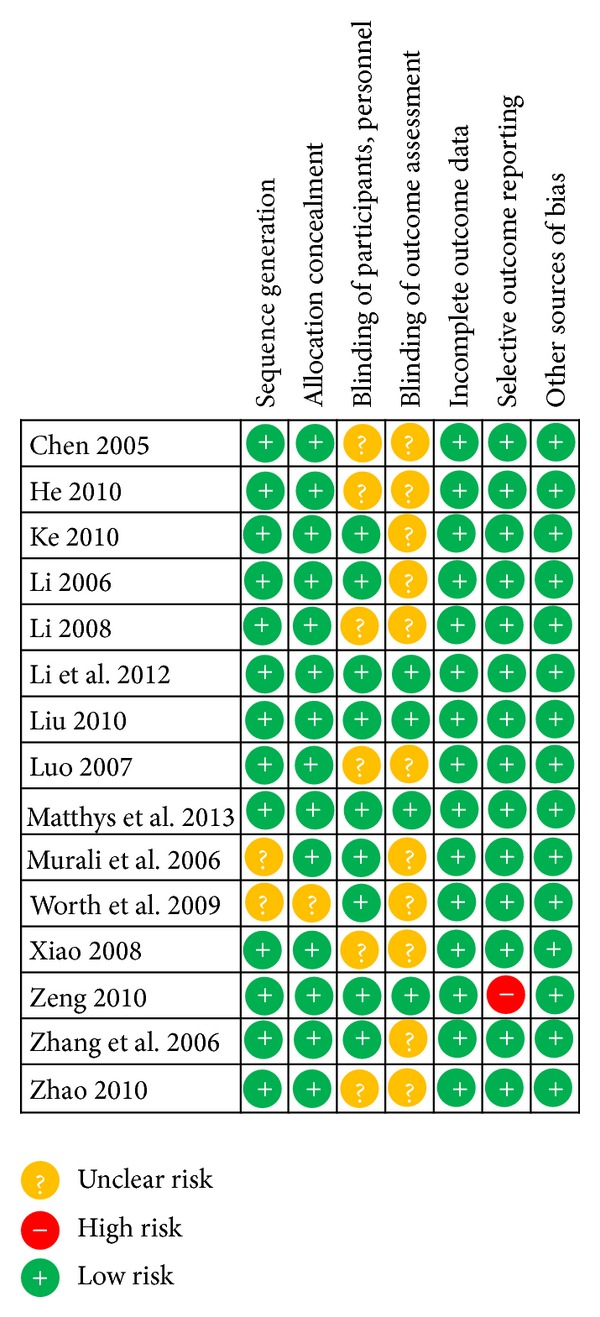
Risk of bias. Risk of bias for the included studies was assessed according to the information that comes from studies at low, unclear, or high risk of bias for each item in the risk of bias tool.

**Figure 2:**
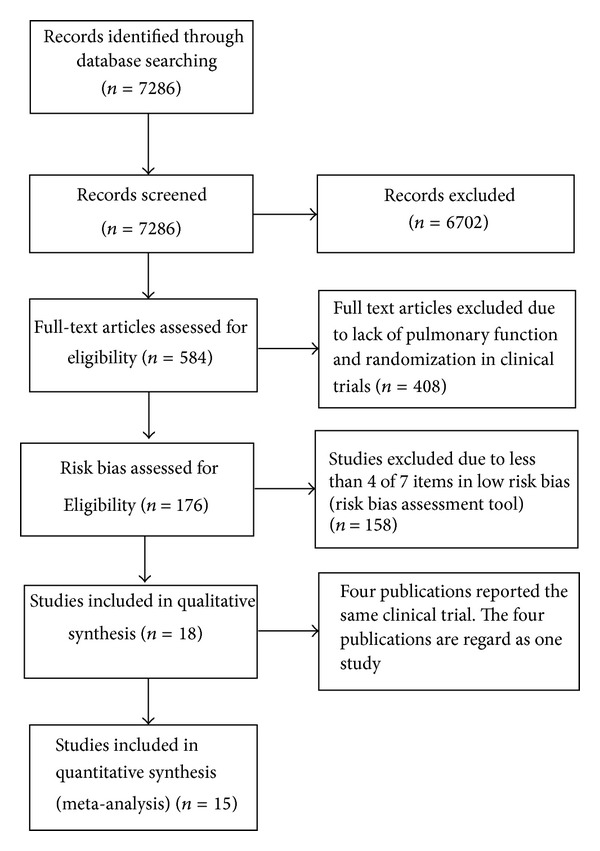
Flow diagram on the screening of study. Fifteen of the studies with high quality were meta-analyzed.

7286 publications and theses were screened by the abstracts and retrieved from databases by two individual reviewers (HC and CM). 6702 publications were excluded while 584 full articles were further assessed for eligibility. 408 publications were excluded due to the lack of data in pulmonary function and randomization in clinical trials. 176 of the studies that satisfied the criteria were included for further analysis. Risk bias of these studies was assessed by seven features including the sequence generation, allocation concealment, blinding of participants and personnel, blinding of outcome assessment, incomplete outcome data, selective outcome reporting, and other biases. Studies with four or more features judged to have low risk bias were regarded as high quality studies. Eighteen high quality publications were identified according to the risk bias assessment tool (Figure 1). Four studies reported the same clinical trial. Therefore, fifteen studies were meta-analyzed for the pulmonary function, symptoms assessment, and quality of life assessment. The characteristics of 15 high quality studies were shown in Table 1. Flow chart of the included studies was shown in Figure 2.

### 3.2. Commonly Used Herbal Medicines in COPD

**Figure 3:**
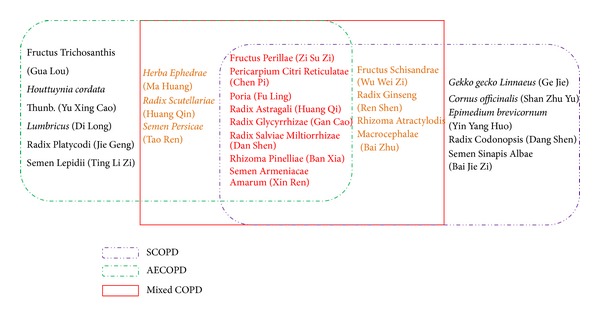
Commonly used herbal medicines from the frequency analysis. The eight Chinese medicines were overlapped in AECOPD and SCOPD, which were further confirmed in the mixed COPD studies.

To identify the commonly used HMs in COPD, we counted the frequency of herb medicines utilized in the 176 clinical trials (Table S1; see Supplementary Material available online at http://dx.doi.org/10.1155/2014/925069). Commonly used HMs for AECOPD and SCOPD were identified as shown in Figure 3. Among commonly used herb medicines, eight herbs including Fructus Perillae (Zi Su Zi), Pericarpium Citri Reticulatae (Chen Pi), Poria (Fu Ling), Radix Astragali (Huang Qi), Radix Glycyrrhizae (Gan Cao), Radix Salviae Miltiorrhizae (Dan Shen), Rhizoma Pinelliae (Ban Xia), and Semen Armeniacae Amarum (Xin Ren) were always used for both AECOPD and SCOPD. To further confirm the findings, fourteen commonly used herbal medicines were identified in the clinical studies without indicating acute exacerbation or stable stage in COPD (mixed COPD). Interestingly, the aforementioned eight most frequently used HMs emerged again. In addition, six other HMs including Herba Ephedrae (Ma Huang), Radix Scutellariae (Huang Qin), Semen Persicae (Tao Ren), Fructus Schisandrae (Wu Wei Zi), Radix Ginseng (Ren Shen), and Rhizoma Atractylodis Macrocephalae (Bai Zhu) used in studies were also found commonly prescribed in AECOPD and SCOPD. Specifically, Herba Ephedrae (Ma Huang), Radix Scutellariae (Huang Qin), and Semen Persicae (Tao Ren) were among the highly frequently used herbs for AECOPD studies, whereas Fructus Schisandrae (Wu Wei Zi), Radix Ginseng (Ren Shen), and Rhizoma Atractylodis Macrocephalae (Bai Zhu) were identified as the highly frequently used herbs for SCOPD.

### 3.3. Pulmonary Function

**Figure 4:**
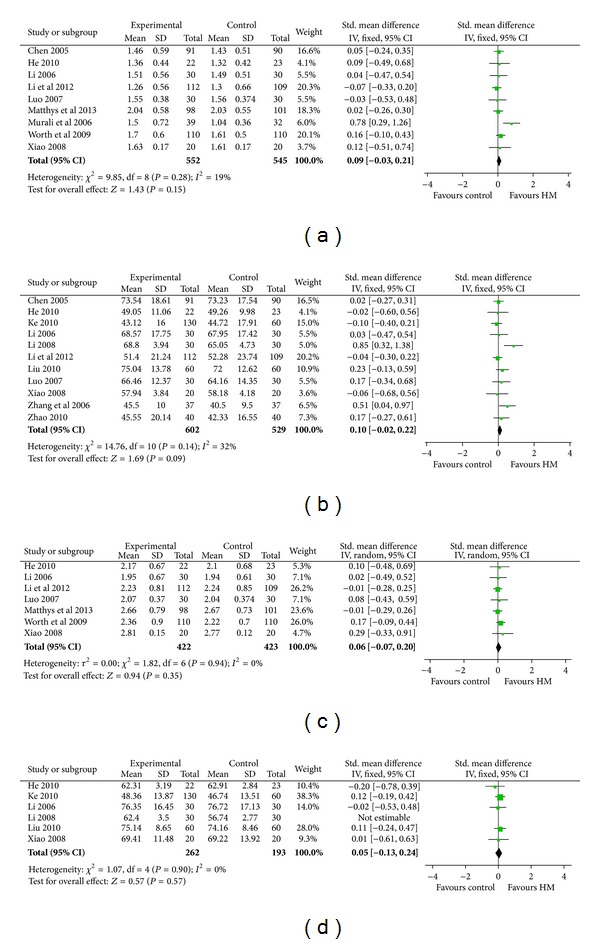
Pulmonary function tests. (a) FEV_1_. (b) FEV%. (c) FVC. (d) FEV/FVC. The pulmonary function test analysis showed that HM had no advantage on improving pulmonary function compared to WM.

Next, we conducted meta-analysis for the included 15 studies [11, 14, 23, 26–40]. Nine studies [14, 26, 27, 29, 31, 34–37] reported the forced expiratory volume in 1 second (FEV_1_) and showed that HM as an adjunct therapy had no advantage on improving FEV_1_ compared to WM (overall effect: *P* = 0.18, heterogeneity:* I* ^2^ = 0%, Figure 4(a)), whereas the baseline of two groups had no significant difference (overall effect: *P* = 1.00, heterogeneity:* I* ^2^ = 0%, Table S2). Eleven high quality studies [26–31, 33, 34, 37, 39, 40] reported FEV% and showed that HM as an adjunct therapy had no advantage on improving FEV% compared with WM (overall effect: *P* = 0.10, heterogeneity:* I* ^2^ = 32%) as shown in Figure 4(b), whereas the baseline of two groups had no significant difference in FEV% (overall effect: *P* = 0.26, heterogeneity:* I* ^2^ = 0%, Table S2). Seven studies [14, 27, 29, 31, 34, 35, 37] reported the forced vital capacity (FVC); pooled data showed that HM as an adjunct therapy had no advantage on improving FVC compared to WM (overall effect: *P* = 0.35, heterogeneity:* I* ^2^ = 0%, Figure 4(c)), whereas the baseline of two groups had no significant difference in FVC (overall effect: *P* = 0.42, heterogeneity:* I* ^2^ = 0%) as shown in Table S2.

Finally, analysis of FEV_1_/FVC in six high quality studies [27–30, 33, 37] consistently showed that HM as an adjunct therapy had no advantage of improving FEV_1_/FVC compared to WM (overall effect: *P* = 0.23; heterogeneity:* I* ^2^ = 83%). The heterogeneity is due to the discrepancy of Li study [30]. After dropping the latter study, the result was in line with the previous finding that HM as an adjunct therapy had no advantage compared to WM (overall effect: *P* = 0.57; heterogeneity: *P* = 0.90) shown in Figure 4(d).

### 3.4. Arterial Blood Gas

Five high quality studies [26, 29, 34, 36, 39] reported the blood gas data. Analysis of blood gas showed that HM as an adjunct therapy had an advantage on reducing PaCO_2_ and increasing PaO_2_. However, there was significant heterogeneity among the studies. Subgroup analysis also indicated the efficacy of the adjunct HM for SCOPD, and AECOPD remains inconclusive due to the heterogeneity of studies (Figure S1).

### 3.5. Symptoms Assessment

**Figure 5:**
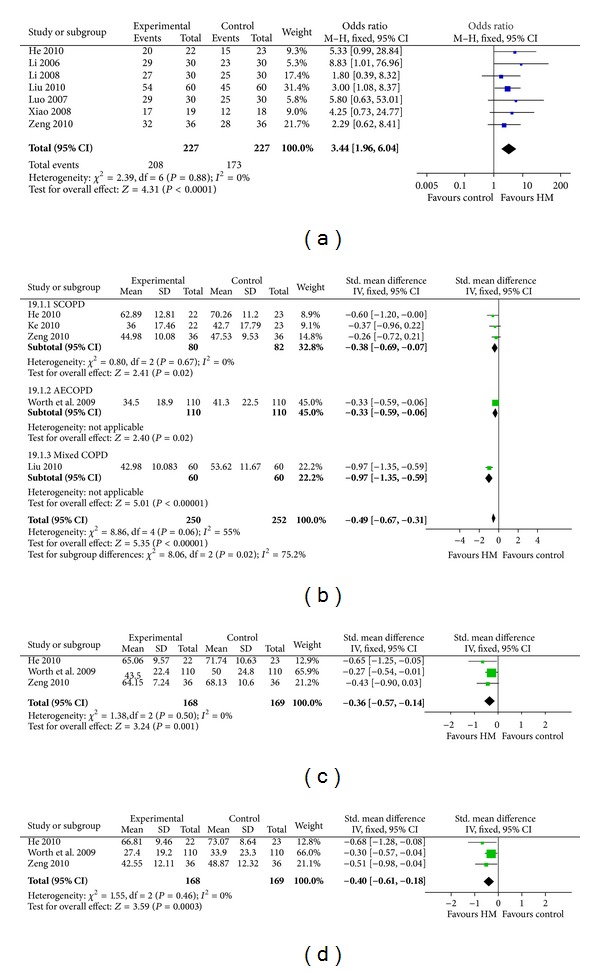
Efficacy and quality of life evaluation. (a) Efficacy of symptom improvement. (b) Subgroup analysis of total score of SGRQ. (c) Activity score of SGRQ. (d) Impact score of SGRQ. Data analysis showed that HM improved the efficacy and quality of life compared to WM. SGRQ, St. George's Respiratory Questionnaire.

Studies reported symptom improvement according to SFDA Guidelines on Clinical Research of TCM New Drugs [41]. The improvement (≥30%) of clinical symptoms and Chinese medicine symptoms was regarded as effective treatment outcome. The pooled analysis of 7 studies [27, 29, 30, 33, 34, 37, 38] showed that HM as an adjunct therapy significantly improved the clinical symptoms compared to WM (overall effect: *P* < 0.00001, heterogeneity:* I* ^2^ = 0%) as shown in Figure 5(a).

### 3.6. Quality of Life Assessment

Five studies reported the findings using St. George's Respiratory Questionnaire (SGRQ), including 3 SCOPD studies [27, 28, 38], 1 AECOPD study [14], and 1 mixed COPD study [33]. As shown in Figure 5(b), subgroup analysis showed that HM as an adjunct therapy had advantage on improving the total score of SGRQ in SCOPD patients compared to WM (overall effect: *P* = 0.02, heterogeneity:* I* ^2^ = 0%). Of these high quality studies, three studies [14, 27, 38] reported symptoms, activities, and impacts scores of SGRQ. Further analysis showed that activity and impact scores of SGRQ in the adjunct HM were superior compared to WM (overall effect: *P* = 0.001, heterogeneity:* I* ^2^ = 0%; overall effect: *P* = 0.0003, heterogeneity:* I* ^2^ = 0%, resp.) (Figures 5(c) and 5(d)), while the efficacy of the adjunct HM in symptom score remains to be further determined due to heterogeneity in the studies (overall effect: *P* = 0.23, heterogeneity:* I* ^2^ = 82%). The baseline TS, activity, impact, and symptom scores were comparable (overall effect: *P* = 0.22, heterogeneity:* I* ^2^ = 0%; overall effect: *P* = 0.30, heterogeneity:* I* ^2^ = 0%; overall effect: *P* = 0.35, heterogeneity:* I* ^2^ = 0%; overall effect: *P* = 0.70, heterogeneity:* I* ^2^ = 0%, resp.) as shown in Table S2.

### 3.7. Subgroup Analysis in Classification of COPD, the Route of HM Administration, and in Placebo/Nonplacebo Groups

We examined the outcome measures with subgroup analysis (AECOPD, SCOP, and Mixed COPD). All outcomes measured show no significant difference among the subgroups except for the SGRQ total score (*P* = 0.02) and PaCO_2_ (*P* < 0.00001) (Table S4). Therefore the SGRQ total score and PaCO_2_ were further analyzed with subgroup analysis. Subgroup analysis for the total score of SGRQ was conducted above (Figure 5(b)). We also found that subgroup analysis of PaCO_2_ measurements indicating the significant heterogeneity among the AECOPD (*P* = 0.002) and data was not pooled in SCOPD studies as only one study reported PaCO_2_. The role of the adjunct HM in improving PaCO_2_ remains inclusive for both SCOPD and AECOPD patients.

In the subgroup analysis with the HM administration route, only one study administrated HM in the route of injection [39], while the left used oral administration. The outcomes involved in the study were FEV%, PaCO_2_, and PO_2_. In a sensitivity test with dropping the study, no significant changes were found in the three outcomes (FEV%, PaCO_2_, and PO_2_) (data was not shown).

We further conducted the subgroup analysis in placebo/nonplacebo groups. All outcomes measurements showed no significant difference except for the SGRQ symptom. Subgroup analysis of SGRQ symptom showed the boarding significant differences between placebo and nonplacebo group (Table S4). Further analysis showed that the subgroup difference may be one of the causes leading to the heterogeneity of studies in the meta-analysis of SGRQ symptoms. However, the subgroup analysis also indicated that the efficacy of the adjunct HM in SGRQ symptom score remains to be further determined. Therefore, subgroup analysis for all outcomes based on placebo and nonplacebo groups did not alter the results in the present study.

## 4. Discussion

COPD is one of the leading causes of morbidity and mortality worldwide; its economic and social burdens are substantial and increasing [42–44]. Several systematic reviews have shown the therapeutic potential of herbal medicine for COPD [18–24]. However, no systematic review was conducted using the pulmonary function as the primary outcome. The present study provided the updated information on the efficacy of HM treatment for COPD using the pulmonary function as the primary outcome measurement, also including arterial blood gas, clinical symptoms, and SGRQ measurements.

Pulmonary function is the essential criteria for the diagnosis of COPD according to GOLD [1]. We found that pooled studies revealed that HM as an adjunct therapy had no advantage on improving the pulmonary function (FEV_1_, FEV%, FVC, and FEV_1_/FVC) in these high quality studies. In contrast, many studies in low quality claimed the improvement of pulmonary function after HM treatment. Interestingly, the pooled arterial blood gas test showed an improvement of PaO_2_ and PaCO_2_ in COPD patients, but it remains inclusive due to the heterogeneity among studies. In addition, lung function impairment in Stages I and II of SCOPD patients is not the most significant concern. But we did not exclude these patients in the meta-analysis due to lack of data from publications. The discrepancies need to be further clarified using a large scale, well-designed clinical trial and using the pulmonary function as the primary outcome.

Quality of life has become an important outcome measure in COPD patients since COPD is a long term disease without cure. SGRQ was widely used for QoL assessment in COPD patients within 3 categories (activity, symptoms, and impact). Analysis of SGRQ from high quality studies showed that HM as an adjunct therapy had an advantage over WM on total score, activity score, and impact score. However, the efficacy of HM as an adjunct therapy in improving the symptoms remains unclear due to the presence of heterogeneity. Importantly, the efficacy assessment of clinical symptoms and Chinese medicine symptoms partly covers symptoms assessed in SGRQ. Therefore, HM as an adjunct therapy may have advantage on improving quality of life and symptom compared to WM.

In the study, eight HMs frequently used for COPD were identified from 176 studies. The HMs fully follow the principles of Chinese medicine prescription for treating AECOPD and SCOPD. Furthermore, we found that the identified Chinese medicines have functions of regulating qi to reduce phlegm (e.g., Pericarpium Citri Reticulatae (Chen Pi), Rhizoma Pinelliae (Ban Xia), Semen Armeniacae Amarum (Xin Ren), and Fructus Perillae (Zi Su Zi)) as well as tonifying qi and promoting blood circulation (Radix Astragali (Huang Qi), Radix Salviae Miltiorrhizae (Dan Shen)). Herba Ephedrae (Ma Huang), Semen Persicae (Tao Ren), and Radix Scutellariae (Huang Qin) have functions such as clearing heat, promoting blood circulation, and facilitating flow in the lung. Radix Ginseng (Ren Shen), Rhizoma Atractylodis Macrocephalae (Bai Zhu), and Fructus Schisandrae (Wu Wei Zi) have the functions of tonifying qi, strengthening spleen to reduce phlegm, and arresting persistent cough. The commonly used herbs for AECOPD and SCOPD in the study were also aligned with the common differentiated symptoms in Chinese medicine for COPD (phlegm-heat in AECOPD and spleen and kidney deficiency in SCOPD).

Moreover, the usage of these herbs was also in line with the biological actions of herbs discovered by modern science. COPD is characterized by a chronic inflammation in the pulmonary tissue [45]. It has been shown that Semen Armeniacae Amarum (Xin Ren) reduced airway inflammation and selectively inhibited the type 2 helper T cell responses [46]. Amygdalin, the active compound from Semen Armeniacae Amarum (Xin Ren), has the high affinity for *β*2-Adrenoceptor [47] and may act as an agonist to induce bronchodilation in COPD patients. Similarly, Fructus Perillae (Zi Su Zi) has been shown to be anti-inflammatory in airway and to restore the Th1/Th2 imbalance in the immune system [48]. Radix Astragali (Huang Qi) has immunologic benefits by stimulating macrophage and natural killer cell activity and inhibiting T-helper cell type 2 cytokines [49].

Pericarpium Citri Reticulatae (Chen Pi), Rhizoma Pinelliae (Ban Xia), Poria (Fu Ling), and Radix Glycyrrhizae (Gan Cao) identified in the study are the components of a famous Chinese medicine formula, namely, Er-Chen decoction, which functions as the removing phlegm in the principle of Chinese medicine. In vitro study shows that the extract of Pericarpium Citri Reticulatae (Chen Pi) has antioxidant and antimicrobial activities [50]. Studies have shown that both Rhizoma Pinelliae (Ban Xia) and Poria (Fu Ling) have sedative effects [51, 52], which may improve the quality of sleep in COPD patients. Moreover, Rhizoma Pinelliae (Ban Xia) inhibits TNF-*α*-induced NF-*κ*B activation [53], and the extraction from Poria (Fu ling) has immunoregulatory effect [54]. In addition, San'ao decoction, a famous Chinese medicine formula composed of three herbs identified in the study, Semen Armeniacae Amarum (Xin Ren), Radix Glycyrrhizae (Gan Cao), and Herba Ephedrae (Ma Huang), has shown anti-inflammation and hyperresponsiveness in airway of a murine model [55].

Though the molecular mechanisms of how HM improves QoL and clinical symptoms of COPD patients remain largely unexplored, multiple effects on anti-inflammation, immunoregulation, sedation, and bronchodilation may be central roles of Chinese medicine prescriptions in the COPD treatment. In addition, the quality control of HMs was not clearly stated in the included studies. The reproducibility of these medications to COPD remains to be further explored.

Analysis of adverse events from the included studies showed that HM as an adjunct therapy has low incidence of adverse effects. But no serious adverse effects were caused by herbal medicines in COPD patients. The common adverse effects are abdominal (“stomach”) discomfort, nausea and vomiting, skin allergy, and pain. It should be pointed out that only one-third of the studies (64 studies/176 studies) had included adverse effect as outcomes. Moreover, studies have shown that herb-drug interaction is a potential risk factor for the adverse events [56–58]. The safety of herbs or herb-drug interaction in COPD treatment needs further investigation.

The quality of clinical trials has to be improved in the future studies. In the present study, 15 studies (18 publications) were identified as high quality studies while others were quasi-RCTs and RCTs with high risk bias. Randomization is one of the major concerns in the clinical trials. Most of the studies only stated that there is “randomization” in them, even when there is a lack of the details about the randomization methods. Descriptions of the methods may minimize or avoid selection bias. Indeed, randomization in some studies we reviewed has not been properly performed; for instance, the patients have been allocated according to the date or the odd and even sequence of admission. Selection bias may also arise from inadequate allocation concealments. In the 176 studies that have been reviewed, only fifteen studies described concealments of allocation. Central allocation or sequentially numbered, opaque, and sealed envelopes are recommended in future studies.

Blinding is the critical method to minimize or avoid the bias, which is absent in studies even in those studies with high quality. Blinding to participants may avoid performance bias. Placebo with similar odor, color, and taste to the intervention should be applied in future studies. It is recommended that there should be an outcome assessment to measure detection bias when TCM practitioners are involved in diagnosing and prescribing for patients.

Data incompetence is also a concern in clinical trials; for instance, few studies reported the details on the dropout or the followup of the study. Side effects or adverse effects should be reported as the outcome of the trials. The clinical trials can follow the international guideline for good clinical practice that has been developed by the International Conference on Harmonization Global Cooperation Group (http://www.ich.org/products/guidelines/efficacy/article/efficacy-guidelines.html). More importantly, it should be pointed out that not all low quality studies are attributed to the low quality of clinical trial design and conduct, but there is also inappropriate or inadequate reporting. As most of the study information comes from publications, the incomplete reporting of clinical trials in articles increases unclear risk of bias. Consolidated Standards of Reporting Trials (CONSORT) group has developed the standard reporting guideline to alleviate the problems arising from inadequate reporting of randomized controlled trials [59].

## 5. Conclusion

Current evidence reveals that HM as an adjunct therapy is uncertain to improve the pulmonary function but may improve clinical symptoms and quality of life for COPD patients. Studies with large scale and double-blind randomized controlled trials are required to confirm the role of HM in the management of COPD.

## Supplementary Material

Table S1: Frequency analysis of herbal medicines.
Table S2: Baseline of included studies.
Table S3: Subgroup differences among AECOPD, SCOPD, and/or Mixed COPD.
Table S4: Subgroup differences between placebo/non-placebo groups.
Supplementary Figure S1 Arterial blood gas: (A). Subgroup analysis of PaO_2_ (B). Subgroup analysis of PaCO_2_. Pooled studies showed HM may improve PaCO_2_ and PaO_2_ for AECOPD, which remains inclusive due the heterogeneity among studies.

## Figures and Tables

**Table 1:** Characteristics of the included studies.

Studies	Diagnosis	Intervention group	Control group	Outcomes	Duration
Chen 2005 [26]	SCOPD/I~II	Chinese herb decoction + WM	WM	Clinical symptom; pulmonary function; arterial blood gas; medical expense; kidney, liver, urine, and blood routine test; and safety examination	6 months
He 2010 [27]	SCOPD/II~III	Chinese herb decoction + WM	WM	Clinical symptom; pulmonary function; and SGRQ	3 months
Ke 2010 [28]	SCOPD/I~IV	Chinese herb decoction + WM	Placebo + WM	Clinical symptom; pulmonary function; SGRQ; 6MWD; BODE; kidney, liver, urine, and blood routine test; and safety examination	2 months
Li 2006 [29]	AECOPD/I~II	Chinese herb decoction + WM	WM	Clinical symptom; pulmonary function; arterial blood gas; kidney, liver, urine, and blood routine test; TNF-*α*; and safety examination	10 days
Li 2008 [30]	SCOPD/I~II	Bufei-Yishen-Huayu decoction + WM	WM	Clinical symptom; pulmonary function; kidney, liver, urine, blood, and stool routine test; safety examination; TNF-*α*; and TGF-*β*1	2 weeks
Li et al. 2012 [11], Xie et al. 2013 [23], Li et al. 2012 [31], and Li et al. 2013 [32]	SCOPD/I~III	Bu-Fei Yi-Shen granule + Shu-Fei Tie ointment + WM	Placebo + WM	Frequency and duration of acute exacerbation of COPD; pulmonary function; quality of life; 6MWD; clinical symptom; and safety examination	4 months
Liu 2010 [33]	NA/NA	Chinese herb decoction + WM	Placebo + WM	Clinical symptom; pulmonary function; SGRQ; and safety examination	1 month
Luo 2007 [34]	AECOPD/I~III	Qinjin-Huatan decoction + WM	WM	Clinical symptom; pulmonary function; kidney, liver, urine, blood, and stool routine test; arterial blood gas; and safety examination	10 days
Matthys et al. 2013 [35]	SCOPD/II~III	EPs 7630 (roots of *Pelargonium sidoides*) + WM	Placebo + WM	Time to first exacerbation of COPD; number and duration of exacerbations; pulmonary function; SGRQ; Integrative Medicine Patient Satisfaction Scale; laboratory safety parameters; and sputum examination	6 months
Murali et al. 2006 [36]	SCOPD/II	Herb (*Bryonia alba*, Cephaelis ipecacuanha, and *Drosera peltata*) extraction + WM	Placebo + WM	Clinical symptom; pulmonary function; arterial blood gas; and safety observation	6 months
Worth et al. 2009 [14]	AECOPD/II~III	Herb (Eucalyptole) extraction + WM	Placebo + WM	Frequency, duration, and severity of exacerbations; pulmonary function; clinical symptom; SGRQ; and safety examination	6 months
Xiao 2008 [37]	SCOPD/I~III	Chinese herb extraction + routine medication	Theophylline + routine medication	Clinical symptom; pulmonary function; and IL-10 and TNF-*α* in the peripheral blood	3 months
Zeng 2010 [38]	SCOPD/I~II	Chinese herb decoction + WM	Placebo + WM	Frequency, duration, and severity of exacerbations; pulmonary function; clinical symptom; SGRQ; and safety examination	4 weeks
Zhang et al. 2006 [39]	AECOPD/NA	Chinese herb injection + WM	Placebo + WM	Clinical symptom; pulmonary function; arterial blood gas; and safety observation	1 week
Zhao 2010 [40]	SCOPD/II~IV	Chinese herb decoction + WM	WM	Clinical symptom; BODE index; 6MWD; sTNF and iNOs; and safety observation	3 months

BODE: BMI, obstruction, dyspnea, and exercise; SGRQ: St. George's Respiratory Questionnaire; WM: western medicine; 6MWD: six-minute walk distance.

## References

[1] Vestbo J, Hurd SS, Agusti AG (2013). Global strategy for the diagnosis, management, and prevention of chronic obstructive pulmonary disease: GOLD executive summary. *American Journal of Respiratory and Critical Care Medicine*.

[2] Rabe KF, Hurd S, Anzueto A (2007). Global strategy for the diagnosis, management, and prevention of chronic obstructive pulmonary disease: GOLD executive summary. *American Journal of Respiratory and Critical Care Medicine*.

[3] Mathers CD, Loncar D (2006). Projections of global mortality and burden of disease from 2002 to 2030. *PLoS Medicine*.

[4] World Health Organization Projections of Mortality and Burden of Disease, 2004–2030. http://www.who.int/healthinfo/global_burden_disease/GBD_report_2004update_full.pdf.

[5] Qaseem A, Wilt TJ, Weinberger SE (2011). Diagnosis and management of stable chronic obstructive pulmonary disease: a clinical practice guideline update from the American College of Physicians, American College of Chest Physicians, American Thoracic Society, and European Respiratory Society. *Annals of Internal Medicine*.

[6] Evensen AE (2010). Management of COPD exacerbations. *American Family Physician*.

[7] McCrory DC, Brown C, Gelfand SE, Bach PB (2001). Management of acute exacerbations of COPD: a summary and appraisal of published evidence. *Chest*.

[8] Hunter MH, King DE (2001). COPD: management of acute exacerbations and chronic stable disease. *American Family Physician*.

[9] Rodriguez-Roisin R (2006). COPD exacerbations · 5: management. *Thorax*.

[10] Ng TP, Niti M, Yap KB, Tan WC (2012). Curcumins-rich curry diet and pulmonary function in Asian older adults. *PLoS ONE*.

[11] Li S-Y, Li J-S, Wang M-H (2012). Effects of comprehensive therapy based on traditional Chinese medicine patterns in stable chronic obstructive pulmonary disease: a four-center, open-label, randomized, controlled study. *BMC Complementary & Alternative Medicine*.

[12] Mukaida K, Hattori N, Kondo K (2011). A pilot study of the multiherb Kampo medicine bakumondoto for cough in patients with chronic obstructive pulmonary disease. *Phytomedicine*.

[13] Gross D, Shenkman Z, Bleiberg B, Dayan M, Gittelson M, Efrat R (2002). Ginseng improves pulmonary functions and exercise capacity in patients with COPD. *Monaldi Archives for Chest Disease*.

[14] Worth H, Schacher C, Dethlefsen U (2009). Concomitant therapy with Cineole (Eucalyptole) reduces exacerbations in COPD: a placebo-controlled double-blind trial. *Respiratory Research*.

[15] Shinozuka N, Tatsumi K, Nakamura A, Terada J, Kuriyama T (2007). The traditional herbal medicine hochuekkito improves systemic inflammation in patients with chronic obstructive pulmonary disease. *Journal of the American Geriatrics Society*.

[16] Tatsumi K, Shinozuka N, Nakayama K, Sekiya N, Kuriyama T, Fukuchi Y (2009). Hochuekkito improves systemic inflammation and nutritional status in elderly patients with chronic obstructive pulmonary disease. *Journal of the American Geriatrics Society*.

[17] Hong M-L, Yang G-Z, Chen W-X, Gao L-Y, Cai S-H, Dai S-Z (2005). Effect of Yufeining on induced sputum interleukin-8 in patients with chronic obstructive pulmonary disease at the stable phase. *Chinese Journal of Integrative Medicine*.

[18] Zhong Y, Mao B, Wang G (2010). Tanreqing injection combined with conventional western medicine for acute exacerbations of chronic obstructive pulmonary disease: a systematic review. *The Journal of Alternative and Complementary Medicine*.

[19] Wu L, Chen Y, Guo X (2013). Oral *Huangqi* formulae for stable chronic obstructive pulmonary disease: a systematic review and meta-analysis. *Evidence-Based Complementary and Alternative Medicine*.

[20] An X, Zhang AL, Yang AW (2011). Oral ginseng formulae for stable chronic obstructive pulmonary disease: a systematic review. *Respiratory Medicine*.

[21] An X, Zhang AL, May BH, Lin L, Xu Y, Xue CC (2012). Oral Chinese herbal medicine for improvement of quality of life in patients with stable chronic obstructive pulmonary disease: a systematic review. *The Journal of Alternative and Complementary Medicine*.

[22] Wu R, Fengjie Z, Li Y (2013). Modified dachengqi decoction combined with conventional treatment for treating acute exacerbation of chronic obstructive pulmonary disease: a systematic review based on randomized controlled trials. *Evidence-Based Complementary and Alternative Medicine*.

[23] Xie Y, Li JS, Yu XQ (2013). Effectiveness of Bufei Yishen Granule combined with acupoint sticking therapy on quality of life in patients with stable chronic obstructive pulmonary disease. *Chinese Journal of Integrative Medicine *.

[24] Gao Z, Liu Y, Zhang J, Upur H (2013). Effect of Jianpi therapy in treatment of chronic obstructive pulmonary disease: a systematic review. *Journal of Traditional Chinese Medicine*.

[25] Guo R, Pittler MH, Ernst E (2006). Herbal medicines for the treatmet of COPD: a systematic review. *European Respiratory Journal*.

[26] Chen XM (2005). *The clinical study of scheme of traditional Chinese medicine treatment for alleviation term of chronic obstructive pulmonary disease [M.S. thesis]*.

[27] He WJ (2010). *The efficacy observation of regulating and reinforcing lung and kidney in stable phase patients with chronic obstructive pulmonary disease [M.S. thesis]*.

[28] Ke XX (2010). *Study on clinical application of lung-spleen correlation theory in the stable phase of chronic obstructive pulmonary disease [M.S. thesis]*.

[29] Li SQ (2006). *Effect of Tnf-alpha in the peripheral blood and clinical research on efficacy of acute exacerbation of chronic obstructive pulmonary disease (AECOPD) with the management of clearing away heat-evil and removing phlegm [M.S. thesis]*.

[30] Li SB (2008). *Efficacy of bufeiyishen huayu on chronie obstruetive pulmonary disease [M.S. thesis]*.

[31] Li J-S, Li S-Y, Yu X-Q (2012). Bu-Fei Yi-Shen granule combined with acupoint sticking therapy in patients with stable chronic obstructive pulmonary disease: a randomized, double-blind, double-dummy, active-controlled, 4-center study. *Journal of Ethnopharmacology*.

[32] Li J-S, Li S-Y, Yu X-Q (2013). The effective evaluation on symptoms and quality of life of chronic obstructive pulmonary disease patients treated by comprehensive therapy based on traditional Chinese medicine patterns. *Complementary Therapies in Medicine*.

[33] Liu Z (2010). *A study on the clinical advantage of traditional Chinese medicine applications “treating different diseases with the same method” for small airway dysfunction [M.S. thesis]*.

[34] Luo LW (2007). *The observation of the clinical effect of Jia Wei Qing Jin Hua Tan Tang on AECOPD therapy [M.S. thesis]*.

[35] Matthys H, Pliskevich DA, Bondarchuk OM, Malek FA, Tribanek M, Kieser M (2013). Randomised, double-blind, placebo-controlled trial of EPs 7630 in adults with COPD. *Respiratory Medicine*.

[36] Murali PM, Rajasekaran S, Paramesh P (2006). Plant-based formulation in the management of chronic obstructive pulmonary disease: a randomized double-blind study. *Respiratory Medicine*.

[37] Xiao HZ (2008). *The observation of curative effect and influence of tumor necrosis factor-*α* and interleukin-10 in stable phase of chronic obstructive pulmonary disease with deficiency of both lung and spleen by Qiangji Jianli oral liquid [M.S. thesis]*.

[38] Zeng SH (2010). *The efficacy assessment of managing body and mind in practising traditional Chinese medicine [M.S. thesis]*.

[39] Zhang W, Sun ZG, Liu JB, Lao WG (2006). A clinical observation of 37 cases using the Tan Re Qing injection for acute exacerbation chronic obstructive pulmonary disease. *New Journal of Traditional Chinese Medicine*.

[40] Zhao HN (2010). *Research on the effect of the method of Xuan Fei Hua Tan Tong Luo on the stable chronic obstructive pulmonary disease [M.S. thesis]*.

[41] Zheng XY (2002). *SFDA Guidelines on Clinical Research of TCM New Drugs*.

[42] Halbert RJ, Isonaka S, George D, Iqbal A (2003). Interpreting COPD prevalence estimates: what is the true burden of disease?. *Chest*.

[43] Simoens S (2010). The economic burden of COPD exacerbations. *Journal of Chronic Obstructive Pulmonary Disease*.

[44] Ramsey SD, Sullivan SD (2003). The burden of illness and economic evaluation for COPD. *European Respiratory Journal*.

[45] Oudijk E-JD, Lammers J-WJ, Koenderman L (2003). Systemic inflammation in chronic obstructive pulmonary disease. *European Respiratory Journal*.

[46] Do J-S, Hwang J-K, Seo H-J, Woo W-H, Nam S-Y (2006). Antiasthmatic activity and selective inhibition of type 2 helper T cell response by aqueous extract of semen armeniacae amarum. *Immunopharmacology and Immunotoxicology*.

[47] Zheng XH, Zhao XF, Yang R, Wang SX, Wei YM, Zheng J (2008). *β*
_2_-adrenoceptor affinity chromatography and its application in the screening of the active compounds from *Semen Armeniacae Amarum*. *Chinese Science Bulletin*.

[48] Yim Y-K, Lee H, Hong K-E (2010). Anti-inflammatory and immune-regulatory effects of subcutaneous perillae fructus extract injections on OVA-induced asthma in mice. *Evidence-Based Complementary and Alternative Medicine*.

[49] McCulloch M, See C, Shu X-J (2006). Astragalus-based Chinese herbs and platinum-based chemotherapy for advanced non-small-cell lung cancer: meta-analysis of randomized trials. *Journal of Clinical Oncology*.

[50] Yi ZB, Yu Y, Liang YZ, Zeng B (2008). *In vitro* antioxidant and antimicrobial activities of the extract of *Pericarpium Citri Reticulatae* of a new Citrus cultivar and its main flavonoids. *LWT: Food Science and Technology*.

[51] Cai T-G, Cai Y (2011). Triterpenes from the fungus poria cocos and their inhibitory activity on nitric oxide production in mouse macrophages *via* blockade of activating protein-1 pathway. *Chemistry & Biodiversity*.

[52] Wu X-Y, Zhao J-L, Zhang M, Li F, Zhao T, Yang L-Q (2011). Sedative, hypnotic and anticonvulsant activities of the ethanol fraction from *Rhizoma Pinelliae* Praeparatum. *Journal of Ethnopharmacology*.

[53] Rozema E, Atanasov AG, Fakhrudin N (2012). Selected extracts of Chinese herbal medicines: their effect on NF-*κ*B, PPAR*α* and PPAR*γ* and the respective bioactive compounds. *Evidence-Based Complementary and Alternative Medicine*.

[54] Jang T-R, Kao M-F, Chen C-H, Hsieh K-C, Lai W-Y, Chen Y-Y (2011). Alleviating effects of dehydration under no hyperthermia on the immunomodulatory response to the polysaccharide fraction from fu-ling (Poria cocos) in male collegiate wrestlers. *Chinese Medical Journal*.

[55] Gu P-C, Fan X-S, Jiang C-X, Xu H-Q, Yu J-H, Tang Y-P (2011). Effect of San'ao Decoction on the airway inflammation and hyperresponsiveness in a murine model of lipopolysaccharide-enhanced asthma. *Chinese Journal of Integrative Medicine*.

[56] Izzo AA, Ernst E (2009). Interactions between herbal medicines and prescribed drugs: an updated systematic review. *Drugs*.

[57] Izzo AA, Ernst E (2001). Interactions between herbal medicines and prescribed drugs: a systematic review. *Drugs*.

[58] Pao L-H, Hu OY-P, Fan H-Y, Lin C-C, Liu L-C, Huang P-W (2012). Herb-drug interaction of 50 Chinese herbal medicines on CYP3A4 activity *in vitro* and *in vivo*. *American Journal of Chinese Medicine*.

[59] Schulz KF, Altman DG, Moher D (2010). CONSORT 2010 statement: updated guidelines for reporting parallel group randomized trials. *Annals of Internal Medicine*.

