# Supplementary material for: A Systematic Review and Meta-Analysis of Herbal Medicine on Chronic Obstructive Pulmonary Diseases
Source: Evid Based Complement Alternat Med. 2014 Mar 26;2014:925069. doi: 10.1155/2014/925069 (PMC3984792; doi:10.1155/2014/925069)
Supplement: Supplementary file 1 — Table S1: Frequency analysis of herbal medicines. Table S2: Baseline of included studies. Table S3: Subgroup differences among AECOPD, SCOPD, and/or Mixed COPD. Table S4: Subgroup differences between placebo/non-placebo groups. Supplementary Figure S1 Arterial blood gas: (A). Subgroup analysis of PaO2 (B). Subgroup analysis of PaCO2. Pooled studies showed HM may improve PaCO2 and PaO2 for AECOPD, which remains inclusive due the heterogeneity among studies. [file 925069.f1.pdf]

Table S1 Frequency analysis of herbal medicines

	AECOPD (70 studies)		SCOPD (66 studies)		Mixed COPD (18 studies)	
No.	Herbs	Freq. %	Herbs	Freq. %	Herbs	Freq. %
1	Radix Scutellariae (Huang Qin)	45.7	Radix Astragali (Huang Qi)	60.6	Radix Glycyrrhizae (Gan Cao)	55.6
2	Rhizoma Pinelliae (Ban Xia)	32.9	Rhizoma Atractylodis Macrocephalae (Bai Zhu)	48.5	Radix Salviae Miltiorrhizae (Dan Shen)	50.0
3	Radix Glycyrrhizae (Gan Cao)	28.6	Poria (Fu Ling)	34.8	Poria (Fu Ling)	50.0
4	Semen Armeniaccae Amarum (Xin Ren)	28.6	Fructus Schisandrae (Wu Wei Zi)	33.3	Pericarpium Citri Reticulatae (Chen Pi)	44.4
5	Fructus Trichosanthis (Gua Lou)	28.6	Radix Ginseng (Ren Shen)	31.8	Radix Ginseng (Ren Shen)	44.4
6	Fructus Perillae (Zi Su Zi)	27.1	Pericarpium Citri Reticulatae (Chen Pi)	27.3	Herba Ephedrae (Ma Huang)	38.9
7	Semen Lepidii (Ting Li Zi)	27.1	Radix Glycyrrhizae (Gan Cao)	27.3	Radix Scutellariae (Huang Qin)	33.3
8	Semen Perisicae (Tao Ren)	25.7	Radix Salviae Miltiorrhizae (Dan Shen)	25.8	Rhizoma Pinelliae (Ban Xia)	33.3
9	Radix Astragali (Huang Qi)	22.9	Rhizoma Pinelliae (Ban Xia)	21.2	Rhizoma Atractylodis Macrocephalae (Bai Zhu)	33.3
10	Radix Salviae Miltiorrhizae (Dan Shen)	21.4	Semen Armeniaccae Amarum (Xin Ren)	21.2	Semen Armeniaccae Amarum (Xin Ren)	33.3
11	Herba Ephedrae (Ma Huang)	20.0	Cornus officinalis (Shan Zhu Yu)	21.2	Radix Astragali (Huang Qi)	27.8
12	Pericarpium Citri Reticulatae (Chen Pi)	20.0	Cekko gecko Linnaeus (Ge Jie)	18.2	Semen Perisicae (Tao Ren)	22.2
13	Poria (Fu Ling)	20.0	Fructus Perillae (Zi Su Zi)	18.2	Fructus Schisandrae (Wu Wei Zi)	22.2
14	Lumbricus (Di Long)	18.6	Radix Codonopsis (Dang Shen)	16.7	Fructus Perillae (Zi Su Zi)	22.2
15	Radix Platycodi (Jie Geng)	18.6	Semen Sinapis Albae (Bai Jie Zi)	16.7		
16	Houttuynia cordata Thunb (Yu Xing Cao)	18.6	Epimedium brevicornum (Yin Yang Huo)	16.7		

Table S2 Baseline of included studies

Outcomes	No. of Studies	Heterogeneity	Overall effect
Pulmonary function			
FEV_1	9	P=1.00; I^2= 0%	Z=0.00 (P=1.00)
FEV%	11	P=0.99; I^2= 0%	Z=1.13 (P=0.26)
FVC	7	P=0.94; I^2= 0%	Z=0.81 (P=0.42)
FEV_1/FVC	6	P=0.72; I^2= 0%	Z=0.61 (P=0.54)
Arterial blood gas			
PaO_2	5	P=0.93; I^2= 0%	Z=0.32 (P=0.75)
$PaCO_2$	4	P=0.99; I^2= 0%	Z=2.03 (P=0.04)
SGRQ			
Total score	5	P=0.96; I^2= 0%	Z=1.23 (P=0.22)
Activity	3	P=0.54; I^2= 0%	Z=1.03 (P=0.30)
Impact	3	P=0.38; I^2= 0%	Z=0.93 (P=0.35)
Symptom	3	P=0.89; I^2= 0%	Z=0.39 (P=0.70)

Table S3 Subgroup differences among AECOPD, SCOPD, and/or Mixed COPD

**Outcomes**	**Subgroup differences (AECOPD, SCOPD, and Mixed COPD)**
Pulmonary function	
FEV_1	P=0.82; I^2= 0%
FEV%	P=0.33; I^2= 9.1%
FVC	P=0.44; I^2= 0%
FEV_1/FVC	P=0.45; I^2= 0%
Arterial blood gas	
PaO_2	P=0.89; I^2= 0%
$PaCO_2$	P<0.00001; I^2= 95.9%*
SGRQ	
Total score	P=0.02; I^2= 75.2%*
Activity	P=0.30; I^2= 6.9%
Impact	P=0.24; I^2= 26.7%
Symptom	P=0.97; I^2= 76.5%
Symptoms Assessment	P=0.57; I^2= 0%

* indicates significant difference among subgroups.

Table S4 Subgroup differences between placebo/non-placebo groups

**Outcomes**	**Subgroup differences (placebo vs non-placebo group)**
Pulmonary function	
FEV_1	P=0.50; I^2= 0%
FEV%	P=0.75; I^2= 0%
FVC	P=0.73; I^2= 0%
FEV_1/FVC	P=0.56; I^2= 0%
Arterial blood gas	
PaO_2	P=0.51; I^2= 0%
$PaCO_2$	P=0.89; I^2= 0%
SGRQ	
Total score	P=0.75; I^2= 0%
Activity	P=0.31; I^2= 3.9%
Impact	P=0.32; I^2= 0%
Symptom	P=0.04; I^2= 76.5%*
Symptoms Assessment	P=0.43; I^2= 0%

* indicates significant difference between subgroups.

Supplementary Figure S1 Arterial blood gas

A. PaO_2

Study or Subgroup	Experimental Mean	Experimental SD	Experimental Total	Control Mean	Control SD	Control Total	Weight	Std. Mean Difference IV, Random, 95% CI
**19.5.1 AECOPD**								
Li 2006	86.37	3.86	30	79.53	3.81	30	18.9%	1.76 [1.16, 2.36]
Luo 2007	80.87	21.1	30	76.67	10.37	30	19.9%	0.25 [-0.26, 0.76]
Zhang et al 2006	76.5	11.2	37	69.5	10.8	37	20.2%	0.63 [0.16, 1.10]
**Subtotal (95% CI)**			**97**			**97**	**59.0%**	**0.86 [0.04, 1.68]**
Heterogeneity: Tau² = 0.45; Chi² = 14.78, df = 2 (P = 0.0006); I² = 86%								
Test for overall effect: Z = 2.06 (P = 0.04)								
**19.5.2 SCOPD**								
Chen 2005	81.525	7.65	91	79.95	6.225	90	21.6%	0.22 [-0.07, 0.52]
Murali et al 2006	92.03	0.76	39	81.8	8.39	32	19.4%	1.79 [1.23, 2.35]
**Subtotal (95% CI)**			**130**			**122**	**41.0%**	**0.99 [-0.54, 2.52]**
Heterogeneity: Tau² = 1.17; Chi² = 23.79, df = 1 (P < 0.00001); I² = 96%								
Test for overall effect: Z = 1.26 (P = 0.21)								
**Total (95% CI)**			**227**			**219**	**100.0%**	**0.91 [0.25, 1.56]**
Heterogeneity: Tau² = 0.49; Chi² = 39.69, df = 4 (P < 0.00001); I² = 90%								
Test for overall effect: Z = 2.72 (P = 0.007)								
Test for subgroup differences: Chi² = 0.02, df = 1 (P = 0.89), I² = 0%								

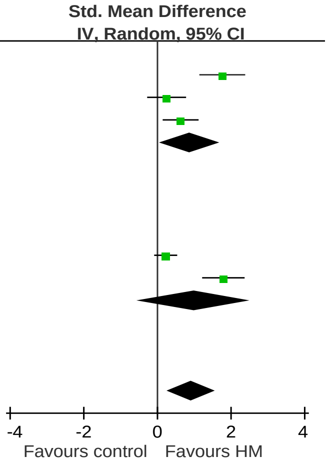

B. $PaCO_2$

Study or Subgroup	Experimental Mean	Experimental SD	Experimental Total	Control Mean	Control SD	Control Total	Weight	Std. Mean Difference IV, Random, 95% CI
**19.3.1 AECOPD**								
Li 2006	42.58	2.87	30	57.32	5.07	30	24.2%	-3.53 [-4.36, -2.70]
Luo 2007	48.4	6.11	30	64.73	10.64	30	25.0%	-1.86 [-2.47, -1.25]
Zhang et al 2006	48.5	11.5	37	70	11	37	25.1%	-1.89 [-2.44, -1.34]
**Subtotal (95% CI)**			**97**			**97**	**74.3%**	**-2.38 [-3.32, -1.44]**
Heterogeneity: Tau² = 0.57; Chi² = 12.37, df = 2 (P = 0.002); I² = 84%								
Test for overall effect: Z = 4.97 (P < 0.00001)								
**19.3.2 SCOPD**								
Chen 2005	49.8	4.8	91	49.2	6.525	90	25.7%	0.10 [-0.19, 0.40]
**Subtotal (95% CI)**			**91**			**90**	**25.7%**	**0.10 [-0.19, 0.40]**
Heterogeneity: Not applicable								
Test for overall effect: Z = 0.70 (P = 0.48)								
**Total (95% CI)**			**188**			**187**	**100.0%**	**-1.77 [-3.33, -0.21]**
Heterogeneity: Tau² = 2.44; Chi² = 105.32, df = 3 (P < 0.00001); I² = 97%								
Test for overall effect: Z = 2.22 (P = 0.03)								
Test for subgroup differences: Chi² = 24.53, df = 1 (P < 0.00001), I² = 95.9%								

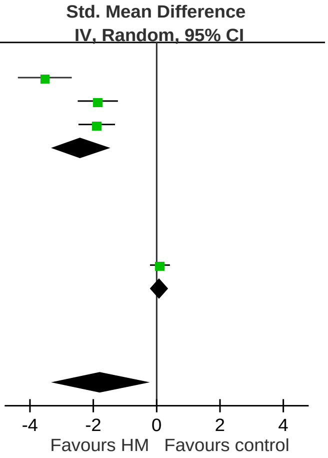

Supplementary Figure S1 Arterial blood gas. (A). Subgroup analysis of PaO_2 (B). Subgroup analysis of $PaCO_2$. Pooled studies showed HM may improve $PaCO_2$ and PaO_2 for AECOPD, which remains inclusive due the heterogeneity among studies.
